# 3,5-Dimethyl-1-(2-pyridylcarbon­yl)-5-[(2-pyridylcarbon­yl)hydrazino]-2-pyrazoline methanol hemisolvate

**DOI:** 10.1107/S1600536808027335

**Published:** 2008-08-30

**Authors:** Yuting Chen, Hua Yang, Dacheng Li, Daqi Wang

**Affiliations:** a Department of Chemistry, Dezhou University, Dezhou 253023, People’s Republic of China; b College of Chemistry and Chemical Engineering, Liaocheng University, Liaocheng 252059, People’s Republic of China

## Abstract

The title compound, C_17_H_18_N_6_O_2_·0.5CH_3_OH, exists in the double keto form and adopts a highly puckered geometry, stabilized by intra­molecular N—H⋯O and N—H⋯N hydrogen bonds. Inter­molecular N—H⋯N hydrogen bonds and π–π stacking inter­actions [centroid–centroid separation = 3.654 (1) Å] assemble the mol­ecules into chains running in the [111] direction. The methanol solvent mol­ecule is disordered over two sites related by inversion and forms a bifurcated O—H⋯(N,O) hydrogen bond.

## Related literature

Two manganese metallocrowns with *N*-acyl-3-hydr­oxy-2-naphthalenecarbohydrazide ligands were synthesized by Dou *et al.* (2006 ▶). The 1-benzoyl-3,5-dimethyl-5-(1-benzoyl­hydrazido)pyrazoline ligand and two pyrazolone derivatives were synthesized by Liu *et al.* (2004 ▶) and Mukhopadhyay & Pal (2004 ▶).
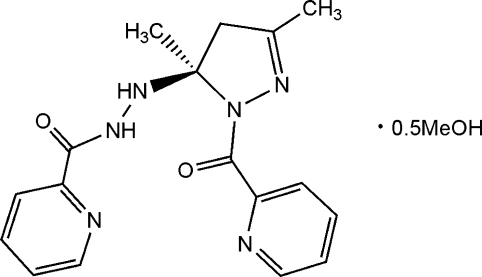


## Experimental

### 

#### Crystal data

C_17_H_18_N_6_O_2_·0.5CH_4_O
*M* *_r_* = 354.40
Triclinic, 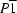

*a* = 9.0111 (12) Å
*b* = 10.6406 (17) Å
*c* = 10.814 (2) Å
α = 78.221 (1)°
β = 66.414 (1)°
γ = 86.477 (2)°
*V* = 930.0 (2) Å^3^
*Z* = 2
Mo *K*α radiation
μ = 0.09 mm^−1^
*T* = 298 (2) K
0.53 × 0.48 × 0.46 mm

#### Data collection

Bruker SMART1000 CCD diffractometer
Absorption correction: multi-scan (*SADABS*; Sheldrick, 1996 ▶) *T*_min_ = 0.955, *T*_max_ = 0.960
4781 measured reflections
3189 independent reflections
2189 reflections with *I* > 2σ(*I*)
*R*_int_ = 0.023

#### Refinement

*R*[*F* ^2^ > 2σ(*F* ^2^)] = 0.065
*wR*(*F* ^2^) = 0.216
*S* = 1.00
3189 reflections
245 parameters
H-atom parameters constrained
Δρ_max_ = 0.84 e Å^−3^
Δρ_min_ = −0.20 e Å^−3^

### 

Data collection: *SMART* (Siemens, 1996 ▶); cell refinement: *SAINT* (Siemens, 1996 ▶); data reduction: *SAINT*; program(s) used to solve structure: *SHELXS97* (Sheldrick, 2008 ▶); program(s) used to refine structure: *SHELXL97* (Sheldrick, 2008 ▶); molecular graphics: *SHELXTL* (Sheldrick, 2008 ▶); software used to prepare material for publication: *SHELXTL*.

## Supplementary Material

Crystal structure: contains datablocks global, I. DOI: 10.1107/S1600536808027335/hb2780sup1.cif

Structure factors: contains datablocks I. DOI: 10.1107/S1600536808027335/hb2780Isup2.hkl

Additional supplementary materials:  crystallographic information; 3D view; checkCIF report

## Figures and Tables

**Table 1:** Hydrogen-bond geometry (Å, °)

*D*—H⋯*A*	*D*—H	H⋯*A*	*D*⋯*A*	*D*—H⋯*A*
N3—H3⋯N6	0.88	2.30	2.667 (3)	105
N3—H3⋯N4^i^	0.88	2.50	3.136 (3)	130
N4—H4⋯O1	0.88	2.59	3.167 (3)	124
N4—H4⋯O2	0.88	2.33	2.727 (3)	108
O3—H3*A*⋯O2	0.82	2.56	2.932 (9)	109
O3—H3*A*⋯N4	0.82	2.62	3.361 (9)	151

Symmetry code: (i) 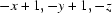.

**Fig. 1.**
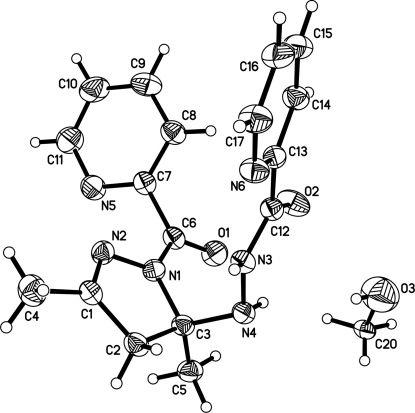
The molecular structure of (I). Displacement ellipsoids for the non-hydrogen atoms are drawn at the 30% probability level.

**Fig. 2.**
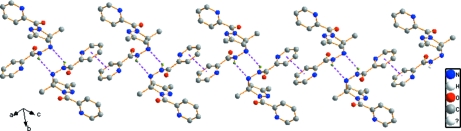
Crystal packing of (I); symmetry code: (i) 1 - x, 1 - y, -z; (ii) -x, 1 - y, 1 - z.

## supplementary crystallographic information

### Comment

After assembling successfully two azametallacrowns based on *N*-acyl-3-hydroxy-2-naphthalenecarbohydrazide (Dou *et al.*, 2006), and as an extension of our work on the structures of aroylhydrazine derivatives, the title compound, (I), was synthesized and characterized.

In compound (I), the C6—O1 and C12—O2 distances are 1.225 (3) Å, 1.227 (3)Å respectively, indicating that the molecule of (I) exists in the double keto form (Liu *et al.*, 2004), and the distances of N(1)—N(2), C(3)—N(1) and C(1)—N(2) are 1.399 (3) Å, 1.492 (3)Å and 1.266 (3)Å (Table 1), which are in agreement with these of analogous compounds (Mukhopadhyay & Pal, 2004).   The title molecule is chiral: in the arbitrarily chosen asymmetric unit, C3 has R configuration, but crystal symmetry generates a racemic mixture.

The three rings in (I), 2-picoloyl ring (A), pyrazoline ring (B) and 2-picoloylhydrazido ring (C), make dihedral angles of 65.5 (2)(A/B), 56.4 (1)(B/C) and 95.5 (1)° (A/C) respectively, showing the whole molecule exhibits highly puckered geometry.

There is intramolecular N4—H4···O1 hydrogen bond, which further stabilizes the molecular configuration (Fig. 1, Table 1), whereas double intermolecular N3—H3···N4 hydrogen bonds link molecules into centrosymmetric dimers. The dimers are assembled into chains along [111] through intermolecular π–π interactions between pyridine rings with a centrosymmetric-centrosymmetric distance of 3.654 (1)Å [*Cg* is a centroid of N6/C13—C17; symmetry code: (ii) -*x*, 1 - *y*, 1 - *z*] (Fig. 2).

### Experimental

2-Picoloylhydrazine (0.548 g, 4 mmol) and ice acetic acid (0.48 g, 8 mmol) were added to a mixture of methanol/glycol (25 ml, 3:2), then 0.21 ml of acetylacetone (0.205 g, 2.05 mmol) was added and reacted for 3 h at 323–333 K. The solution was cooled to room temperature. After the solution was allowed to stand for three weeks, colourless blocks of (I) were obtained.   When recrystallised from pure methanol, the same compound arises.  Yield: 0.591 g, 81.5%. m.p.: 585–587 K. Anal. for C_17.5_H_20_N_6_O_2.5_: Calc. C, 59.25; H, 5.64; N, 23.70; Found: C, 59.21; H, 5.48; N, 23.38%.

### Refinement

The methanol solvent molecule is disordered over two adjacent sites related by inversion.

All the H atoms were placed in idealized positions (C-H = 0.93-0.97Å, N-H = 0.88Å, O-H = 0.82Å) and refined as riding with *U*_iso_(H) = 1.2*U*_eq_(carrier) or 1.5U_eq_(methyl C).

The highest difference peak is 0.80Å from C2.

### Crystal data

**Table:**

C_17_H_18_N_6_O_2_·0.5CH_4_O	*Z* = 2
*M**_r_* = 354.40	*F*_000_ = 374
Triclinic, *P*1	*D*_x_ = 1.266 Mg m^−^^3^
Hall symbol: -P 1	Mo *K*α radiation λ = 0.71073 Å
*a* = 9.0111 (12) Å	Cell parameters from 1800 reflections
*b* = 10.6406 (17) Å	θ = 2.5–24.8º
*c* = 10.814 (2) Å	µ = 0.09 mm^−^^1^
α = 78.221 (1)º	*T* = 298 (2) K
β = 66.414 (1)º	Block, colourless
γ = 86.477 (2)º	0.53 × 0.48 × 0.46 mm
*V* = 930.0 (2) Å^3^	

### Data collection

**Table:**

Bruker SMART1000 CCD diffractometer	3189 independent reflections
Radiation source: fine-focus sealed tube	2189 reflections with *I* > 2σ(*I*)
Monochromator: graphite	*R*_int_ = 0.023
*T* = 298(2) K	θ_max_ = 25.0º
ω scans	θ_min_ = 2.5º
Absorption correction: multi-scan(SADABS; Sheldrick, 1996)	*h* = −10→10
*T*_min_ = 0.955, *T*_max_ = 0.960	*k* = −12→7
4781 measured reflections	*l* = −12→12

### Refinement

**Table:**

Refinement on *F*^2^	Secondary atom site location: difference Fourier map
Least-squares matrix: full	Hydrogen site location: inferred from neighbouring sites
*R*[*F*^2^ > 2σ(*F*^2^)] = 0.065	H-atom parameters constrained
*wR*(*F*^2^) = 0.216	*w* = 1/[σ^2^(*F*_o_^2^) + (0.139*P*)^2^ + 0.2337*P*] where *P* = (*F*_o_^2^ + 2*F*_c_^2^)/3
*S* = 1.00	(Δ/σ)_max_ = 0.001
3189 reflections	Δρ_max_ = 0.84 e Å^−^^3^
245 parameters	Δρ_min_ = −0.20 e Å^−^^3^
Primary atom site location: structure-invariant direct methods	Extinction correction: none

### Special details

**Table:**

Geometry. All e.s.d.'s (except the e.s.d. in the dihedral angle between two l.s. planes) are estimated using the full covariance matrix. The cell e.s.d.'s are taken into account individually in the estimation of e.s.d.'s in distances, angles and torsion angles; correlations between e.s.d.'s in cell parameters are only used when they are defined by crystal symmetry. An approximate (isotropic) treatment of cell e.s.d.'s is used for estimating e.s.d.'s involving l.s. planes.
Refinement. Refinement of *F*^2^ against ALL reflections. The weighted *R*-factor *wR* and goodness of fit *S* are based on *F*^2^, conventional *R*-factors *R* are based on *F*, with *F* set to zero for negative *F*^2^. The threshold expression of *F*^2^ > σ(*F*^2^) is used only for calculating *R*-factors(gt) *etc*. and is not relevant to the choice of reflections for refinement. *R*-factors based on *F*^2^ are statistically about twice as large as those based on *F*, and *R*- factors based on ALL data will be even larger.

### Fractional atomic coordinates and isotropic or equivalent isotropic displacement parameters (Å^2^)

**Table:**

	*x*	*y*	*z*	*U*_iso_*/*U*_eq_	Occ. (<1)
N1	0.4874 (3)	0.8072 (2)	0.1320 (2)	0.0433 (6)	
N2	0.3319 (3)	0.8492 (2)	0.1473 (2)	0.0466 (6)	
N3	0.4382 (3)	0.5456 (2)	0.1581 (2)	0.0433 (6)	
H3	0.3688	0.5191	0.1298	0.052*	
N4	0.5844 (3)	0.6043 (2)	0.0554 (2)	0.0430 (6)	
H4	0.6572	0.6046	0.0905	0.052*	
N5	0.4085 (4)	0.9947 (2)	0.3280 (3)	0.0636 (8)	
N6	0.1726 (3)	0.4050 (2)	0.3258 (2)	0.0508 (6)	
O1	0.6856 (3)	0.7836 (2)	0.2105 (2)	0.0618 (6)	
O2	0.5370 (3)	0.5115 (2)	0.3232 (2)	0.0665 (7)	
O3	0.8469 (9)	0.4142 (7)	0.1517 (8)	0.123 (2)	0.50
H3A	0.7709	0.4329	0.1284	0.185*	0.50
C1	0.3048 (3)	0.8290 (3)	0.0467 (3)	0.0457 (7)	
C2	0.4392 (4)	0.7663 (3)	−0.0514 (3)	0.0493 (7)	
H2A	0.4832	0.8217	−0.1416	0.059*	
H2B	0.4026	0.6859	−0.0599	0.059*	
C3	0.5665 (3)	0.7430 (2)	0.0119 (3)	0.0425 (6)	
C4	0.1544 (4)	0.8701 (4)	0.0264 (4)	0.0692 (9)	
H4A	0.0845	0.9090	0.1013	0.104*	
H4B	0.1001	0.7967	0.0232	0.104*	
H4C	0.1813	0.9311	−0.0586	0.104*	
C5	0.7302 (4)	0.8002 (3)	−0.0844 (3)	0.0545 (8)	
H5A	0.7209	0.8906	−0.1139	0.082*	
H5B	0.7718	0.7598	−0.1631	0.082*	
H5C	0.8027	0.7866	−0.0379	0.082*	
C6	0.5483 (3)	0.8179 (3)	0.2250 (3)	0.0441 (7)	
C7	0.4413 (3)	0.8700 (3)	0.3491 (3)	0.0461 (7)	
C8	0.3925 (4)	0.7926 (3)	0.4769 (3)	0.0554 (8)	
H8	0.4203	0.7066	0.4874	0.066*	
C9	0.3014 (5)	0.8454 (4)	0.5893 (3)	0.0711 (10)	
H9	0.2640	0.7951	0.6774	0.085*	
C10	0.2671 (5)	0.9717 (4)	0.5696 (4)	0.0780 (11)	
H10	0.2066	1.0094	0.6444	0.094*	
C11	0.3214 (5)	1.0429 (4)	0.4402 (4)	0.0750 (10)	
H11	0.2969	1.1295	0.4287	0.090*	
C12	0.4278 (3)	0.4976 (2)	0.2862 (3)	0.0423 (6)	
C13	0.2742 (3)	0.4273 (2)	0.3813 (3)	0.0407 (6)	
C14	0.2423 (4)	0.3888 (3)	0.5204 (3)	0.0510 (7)	
H14	0.3164	0.4058	0.5552	0.061*	
C15	0.0991 (4)	0.3252 (3)	0.6047 (3)	0.0587 (8)	
H15	0.0736	0.2988	0.6985	0.070*	
C16	−0.0062 (4)	0.3010 (3)	0.5495 (3)	0.0639 (9)	
H16	−0.1035	0.2567	0.6048	0.077*	
C17	0.0339 (4)	0.3432 (3)	0.4113 (3)	0.0618 (8)	
H17	−0.0397	0.3278	0.3752	0.074*	
C20	0.9726 (6)	0.5000 (5)	0.0745 (5)	0.0401 (12)	0.50
H20A	1.0622	0.4779	0.1024	0.048*	0.50
H20B	0.9384	0.5854	0.0911	0.048*	0.50
H20C	1.0000	0.5000	0.0000	0.048*	

### Atomic displacement parameters (Å^2^)

**Table:**

	*U*^11^	*U*^22^	*U*^33^	*U*^12^	*U*^13^	*U*^23^
N1	0.0482 (13)	0.0436 (13)	0.0418 (12)	0.0024 (10)	−0.0199 (10)	−0.0125 (10)
N2	0.0497 (14)	0.0456 (13)	0.0477 (13)	0.0048 (10)	−0.0211 (11)	−0.0134 (10)
N3	0.0463 (13)	0.0437 (13)	0.0415 (12)	−0.0045 (10)	−0.0194 (10)	−0.0062 (10)
N4	0.0471 (13)	0.0415 (13)	0.0398 (12)	−0.0012 (10)	−0.0173 (10)	−0.0064 (9)
N5	0.090 (2)	0.0486 (15)	0.0555 (15)	0.0058 (13)	−0.0304 (15)	−0.0154 (12)
N6	0.0494 (14)	0.0605 (15)	0.0429 (13)	−0.0054 (11)	−0.0184 (11)	−0.0088 (11)
O1	0.0585 (14)	0.0762 (15)	0.0609 (13)	0.0051 (11)	−0.0320 (11)	−0.0193 (11)
O2	0.0690 (14)	0.0827 (16)	0.0538 (12)	−0.0232 (12)	−0.0345 (11)	0.0024 (11)
O3	0.113 (5)	0.134 (6)	0.111 (5)	−0.005 (4)	−0.041 (4)	−0.007 (4)
C1	0.0533 (16)	0.0413 (15)	0.0450 (15)	0.0001 (12)	−0.0241 (13)	−0.0042 (12)
C2	0.0630 (18)	0.0496 (16)	0.0419 (15)	0.0019 (13)	−0.0281 (14)	−0.0079 (12)
C3	0.0530 (16)	0.0407 (15)	0.0357 (13)	0.0007 (12)	−0.0186 (12)	−0.0094 (11)
C4	0.065 (2)	0.077 (2)	0.074 (2)	0.0058 (17)	−0.0385 (19)	−0.0120 (18)
C5	0.0588 (18)	0.0510 (17)	0.0454 (16)	−0.0047 (14)	−0.0119 (14)	−0.0077 (13)
C6	0.0533 (17)	0.0404 (15)	0.0443 (15)	−0.0027 (12)	−0.0254 (13)	−0.0069 (12)
C7	0.0574 (17)	0.0432 (16)	0.0478 (16)	−0.0011 (12)	−0.0292 (14)	−0.0125 (12)
C8	0.0680 (19)	0.0529 (17)	0.0497 (17)	−0.0040 (14)	−0.0277 (15)	−0.0092 (14)
C9	0.084 (2)	0.080 (3)	0.0473 (18)	−0.0121 (19)	−0.0231 (18)	−0.0111 (17)
C10	0.093 (3)	0.084 (3)	0.059 (2)	0.007 (2)	−0.024 (2)	−0.036 (2)
C11	0.100 (3)	0.061 (2)	0.067 (2)	0.0109 (19)	−0.030 (2)	−0.0286 (18)
C12	0.0486 (15)	0.0418 (15)	0.0406 (14)	0.0003 (11)	−0.0209 (13)	−0.0101 (12)
C13	0.0461 (15)	0.0403 (14)	0.0384 (14)	0.0033 (11)	−0.0183 (12)	−0.0108 (11)
C14	0.0580 (18)	0.0562 (18)	0.0411 (15)	0.0010 (14)	−0.0223 (14)	−0.0089 (13)
C15	0.0604 (19)	0.066 (2)	0.0420 (16)	−0.0015 (15)	−0.0142 (15)	−0.0052 (14)
C16	0.0520 (18)	0.074 (2)	0.0525 (18)	−0.0037 (15)	−0.0081 (15)	−0.0099 (16)
C17	0.0517 (18)	0.078 (2)	0.0546 (18)	−0.0080 (15)	−0.0206 (16)	−0.0092 (16)
C20	0.025 (2)	0.060 (3)	0.033 (2)	0.003 (2)	−0.015 (2)	0.001 (2)

### Geometric parameters (Å, °)

**Table:**

N1—C6	1.350 (3)	C4—H4C	0.9600
N1—N2	1.399 (3)	C5—H5A	0.9600
N1—C3	1.492 (3)	C5—H5B	0.9600
N2—C1	1.266 (3)	C5—H5C	0.9600
N3—C12	1.343 (3)	C6—C7	1.497 (4)
N3—N4	1.417 (3)	C7—C8	1.369 (4)
N3—H3	0.8800	C8—C9	1.375 (5)
N4—C3	1.473 (3)	C8—H8	0.9300
N4—H4	0.8802	C9—C10	1.354 (5)
N5—C7	1.336 (4)	C9—H9	0.9300
N5—C11	1.339 (4)	C10—C11	1.355 (5)
N6—C17	1.333 (4)	C10—H10	0.9300
N6—C13	1.334 (3)	C11—H11	0.9300
O1—C6	1.225 (3)	C12—C13	1.486 (4)
O2—C12	1.227 (3)	C13—C14	1.388 (4)
O3—C20	1.368 (9)	C14—C15	1.367 (4)
O3—H3A	0.8200	C14—H14	0.9300
C1—C2	1.483 (4)	C15—C16	1.367 (5)
C1—C4	1.484 (4)	C15—H15	0.9300
C2—C3	1.539 (4)	C16—C17	1.371 (5)
C2—H2A	0.9700	C16—H16	0.9300
C2—H2B	0.9700	C17—H17	0.9300
C3—C5	1.501 (4)	C20—H20A	0.9700
C4—H4A	0.9600	C20—H20B	0.9700
C4—H4B	0.9600	C20—H20C	0.7426
			
C6—N1—N2	121.2 (2)	O1—C6—C7	120.7 (2)
C6—N1—C3	125.9 (2)	N1—C6—C7	118.0 (2)
N2—N1—C3	112.7 (2)	N5—C7—C8	123.5 (3)
C1—N2—N1	108.6 (2)	N5—C7—C6	116.5 (2)
C12—N3—N4	120.4 (2)	C8—C7—C6	119.7 (3)
C12—N3—H3	119.6	C7—C8—C9	118.2 (3)
N4—N3—H3	117.0	C7—C8—H8	120.9
N3—N4—C3	111.8 (2)	C9—C8—H8	120.9
N3—N4—H4	110.0	C10—C9—C8	118.9 (3)
C3—N4—H4	101.3	C10—C9—H9	120.5
C7—N5—C11	116.4 (3)	C8—C9—H9	120.5
C17—N6—C13	116.6 (2)	C9—C10—C11	119.5 (3)
C20—O3—H3A	109.5	C9—C10—H10	120.2
N2—C1—C2	114.1 (2)	C11—C10—H10	120.2
N2—C1—C4	122.5 (3)	N5—C11—C10	123.4 (3)
C2—C1—C4	123.3 (3)	N5—C11—H11	118.3
C1—C2—C3	104.4 (2)	C10—C11—H11	118.3
C1—C2—H2A	110.9	O2—C12—N3	122.5 (3)
C3—C2—H2A	110.9	O2—C12—C13	122.0 (2)
C1—C2—H2B	110.9	N3—C12—C13	115.5 (2)
C3—C2—H2B	110.9	N6—C13—C14	123.4 (3)
H2A—C2—H2B	108.9	N6—C13—C12	116.6 (2)
N4—C3—N1	111.9 (2)	C14—C13—C12	120.1 (2)
N4—C3—C5	108.3 (2)	C15—C14—C13	118.3 (3)
N1—C3—C5	113.0 (2)	C15—C14—H14	120.9
N4—C3—C2	110.5 (2)	C13—C14—H14	120.9
N1—C3—C2	99.8 (2)	C14—C15—C16	119.1 (3)
C5—C3—C2	113.2 (2)	C14—C15—H15	120.4
C1—C4—H4A	109.5	C16—C15—H15	120.4
C1—C4—H4B	109.5	C15—C16—C17	119.0 (3)
H4A—C4—H4B	109.5	C15—C16—H16	120.5
C1—C4—H4C	109.5	C17—C16—H16	120.5
H4A—C4—H4C	109.5	N6—C17—C16	123.6 (3)
H4B—C4—H4C	109.5	N6—C17—H17	118.2
C3—C5—H5A	109.5	C16—C17—H17	118.2
C3—C5—H5B	109.5	O3—C20—H20A	109.4
H5A—C5—H5B	109.5	O3—C20—H20B	109.4
C3—C5—H5C	109.5	H20A—C20—H20B	108.0
H5A—C5—H5C	109.5	O3—C20—H20C	111.3
H5B—C5—H5C	109.5	H20A—C20—H20C	109.4
O1—C6—N1	121.3 (3)	H20B—C20—H20C	109.4
			
C6—N1—N2—C1	179.7 (2)	O1—C6—C7—N5	111.1 (3)
C3—N1—N2—C1	−4.8 (3)	N1—C6—C7—N5	−70.4 (3)
C12—N3—N4—C3	−113.3 (3)	O1—C6—C7—C8	−64.1 (4)
N1—N2—C1—C2	1.4 (3)	N1—C6—C7—C8	114.4 (3)
N1—N2—C1—C4	−176.0 (3)	N5—C7—C8—C9	1.7 (5)
N2—C1—C2—C3	2.2 (3)	C6—C7—C8—C9	176.6 (3)
C4—C1—C2—C3	179.6 (3)	C7—C8—C9—C10	−1.6 (5)
N3—N4—C3—N1	48.5 (3)	C8—C9—C10—C11	0.7 (6)
N3—N4—C3—C5	173.7 (2)	C7—N5—C11—C10	−0.2 (6)
N3—N4—C3—C2	−61.7 (3)	C9—C10—C11—N5	0.2 (6)
C6—N1—C3—N4	64.0 (3)	N4—N3—C12—O2	6.3 (4)
N2—N1—C3—N4	−111.2 (2)	N4—N3—C12—C13	−174.3 (2)
C6—N1—C3—C5	−58.5 (3)	C17—N6—C13—C14	0.4 (4)
N2—N1—C3—C5	126.3 (2)	C17—N6—C13—C12	−179.3 (3)
C6—N1—C3—C2	−179.0 (2)	O2—C12—C13—N6	−172.3 (3)
N2—N1—C3—C2	5.7 (3)	N3—C12—C13—N6	8.3 (4)
C1—C2—C3—N4	113.4 (2)	O2—C12—C13—C14	8.0 (4)
C1—C2—C3—N1	−4.5 (3)	N3—C12—C13—C14	−171.4 (2)
C1—C2—C3—C5	−124.8 (2)	N6—C13—C14—C15	−0.2 (4)
N2—N1—C6—O1	−178.5 (2)	C12—C13—C14—C15	179.5 (3)
C3—N1—C6—O1	6.6 (4)	C13—C14—C15—C16	0.5 (5)
N2—N1—C6—C7	3.0 (4)	C14—C15—C16—C17	−1.1 (5)
C3—N1—C6—C7	−171.8 (2)	C13—N6—C17—C16	−1.0 (5)
C11—N5—C7—C8	−0.8 (5)	C15—C16—C17—N6	1.3 (5)
C11—N5—C7—C6	−175.8 (3)		

### Hydrogen-bond geometry (Å, °)

**Table:**

*D*—H···*A*	*D*—H	H···*A*	*D*···*A*	*D*—H···*A*
N3—H3···N6	0.88	2.30	2.667 (3)	105
N3—H3···N4^i^	0.88	2.50	3.136 (3)	130
N4—H4···O1	0.88	2.59	3.167 (3)	124
N4—H4···O2	0.88	2.33	2.727 (3)	108
O3—H3A···O2	0.82	2.56	2.932 (9)	109
O3—H3A···N4	0.82	2.62	3.361 (9)	151

Symmetry codes: (i) −*x*+1, −*y*+1, −*z*.
